# Marjorie Budd Lecture, Abdominal Aortic Aneurysms, Trials and Tribulations, Sir Geoffrey Slaney

**Published:** 1987-11

**Authors:** 


### Marjorie Budd Lecture

Marjorie Budd had a vascular anomaly of her lip successfully treated by Profesor Milnes Walker and that was how the Marjorie Budd visiting professorship came to be instituted and why a vascular surgical subject is particularly appropriate.

Professor Slaney reviewed all the cases of abdominal aortic aneurysm he had personally operated on since his first case in 1960 and these totalled 468.

Most aneurysms of the abdominal aorta were below the renal vessels but a few extended above and were a distinct group with especial surgical difficulties. Damage to the 8th and 9th intercostal arteries could lead to paraplegia and there was an 8% incidence in this group. Complications were proportional to the number of anastomoses that needed to be made including the superior mesenteric, caeliac axis and renal vessels. This was 'tiger country' in this series the mortality was 15%, Crawford in the US specialised in this operation and his mortality was 5%.

Once the diagnosis of abdominal aneurysm is made, deferment of surgery carries the risk of acute rupture and this increases the surgical mortality fivefold. In this series 49 patients were considered to be poor risks and surgery was not advised but of these 21 (42%) ultimately ruptured.

Aortography is not a necessary routine and can even be misleading. There should be no hesitation in dividing the left renal vein if necessary, too severe retraction on this can split the cava and this is very difficult to repair. The aneurysm is not resected but opened and the graft placed within it. Very often the bifurcation need not be resected and a straight graft can be used. A moderate degree of dilatation and tortuosity of the iliac vessels does not require their bypass.

A third of all aneurysms treated in the series had ruptured and there was no change in the incidence of acute rupture since the start of the series in 1960. The incidence of ruptured aortic aneurysm in this country is the highest in the Western world, the reason for this is not clear. It can mimic almost any abdominal emergency but 90% have a palpable mass. The mortlity depended very much on the duration of the operation and was as follows–

*Mortality rate and duration of operation*

| | |
|---|---|
| Less than 2 hrs | 20% |
| 2 – 4 hrs | 33% |
| 4 – 5 hrs | 41% |
| More than 5 hrs | 63% |

There were 5 cases of aorto-duodenal fistual and all had melaena, the bleeding can continue for weeks. In 50% of these cases subsequent graft infection followed, removal of the graft with axillary bypass was the best option for this. In aorto-caval fistula the survival rate is similar to that in ordinary rupture. When postoperative renal failure is threatened early dialysis is advisable. In a third of the cases in which the superior mesenteric artery was comprised gut ischaemia occurred and a second look one or two days later is a good idea.

*Results in the whole series since 1960*

| | Totals | Mortlity Rate % |
|---|---|---|
| Elective | 222 | 7.9 |
| Acute | 72 | 16.0 |
| Ruptured | 174 | 42.0 |
| | 468 | |

5 year survival rate was two thirds, which was roughly the life expectation in that age group. It is therefore a very cost effective operation and a better bargain than cancer surgery.

